# The effect of light curing time and intensity on the bond strength and fracture resistance of orthodontic adhesive

**DOI:** 10.34172/joddd.2023.36990

**Published:** 2023-04-03

**Authors:** Omar K. Mohammed, Mohammed T. Younis, Alaa E. Dawood

**Affiliations:** ^1^Department of Orthodontics, Pedodontics and Preventive Dentistry, College of Dentistry, University of Mosul, Mosul, Iraq; ^2^Department of Conservative Dentistry, College of Dentistry, University of Mosul, Mosul, Iraq

**Keywords:** Curing time, Curing intensity, Orthodontic adhesives

## Abstract

**Background.:**

This study aimed to measure the shear bond strength and compressive strength of orthodontic adhesives at different curing times and intensities.

**Methods.:**

Ninety extracted human premolars were used. Orthodontic brackets were bonded on the buccal surface of the teeth with orthodontic adhesive light-cured using VRN-VAFU LED curing light at different curing times (1, 3 and 5 seconds) and intensities (1000, 1600 and 2300 mW/cm^2^ ). A universal testing machine was used to measure the shear bond strength. The ratio of the adhesive remnant and compressive strength of the orthodontic adhesive, at each curing time at the different intensities, were also evaluated. The results were statistically analyzed using one-way analysis of variance followed by Tukey’s test.

**Results.:**

The lowest bond strength values (6.4, 9.9 and 12.6 MPa) were recorded with 1000 mW/ cm^2^ intensity (at all curing times) in comparison with the other intensities (*P*<0.05). Increasing the curing time significantly increased the bond strength of the orthodontic brackets (*P*<0.05), except when the curing time was increased from 3 sec to 5 sec at 1600 mW/cm^2^ intensity. The highest compressive strength values (130.3, 147.1 and 174 MPa) were recorded at 2300 mW/ cm^2^ intensity (at all curing times) compared to the other intensities (*P*<0.05). The highest values of the ratio of the adhesive remnants were recorded at 1000 mW/cm^2^ intensity (at all curing times) compared to the other intensities (*P*<0.05).

**Conclusion.:**

Although, increasing the curing time and\or the curing intensity has a positive effect on the bond strength and compressive strength of the orthodontic adhesive, it might be possible to suggest reducing the curing time of orthodontic adhesive to 1 sec at curing intensity of 2300 mW/cm^2^.

## Introduction

Orthodontic brackets bonding is considered one of the most time-consuming procedures in dentistry.^1^ Consequently, reducing the time of light curing of orthodontic adhesive would improve the treatment outcome and maximize the patients’ comfort and cooperation. The newly introduced light curing devices such as the light-emitting diode (LED) are reported to be capable of achieving acceptable clinical results, low heat generation and reduced light-curing times of 8 to 10 seconds with light intensity of about 1000 mW/cm^2^. ^2-5^ LED light curing devices with higher intensity could be used with even shorter curing time.^6^

It has been reported that LED that produces light intensity of 3200 mW/cm^2^ can achieve close to 90% composite conversion in 3 seconds^7^ (55-65% degree of conversion is clinically acceptable).^8^ A clinical trial has also revealed that high intensity LED could be used for 6 seconds to bond the orthodontic brackets.^9^ A reduction of light-curing time to 3 seconds for bonding orthodontic brackets produces clinically acceptable bond strength.^6^

On the other hand, reducing the light curing time of orthodontic adhesive will affect the degree of conversion and subsequent physical properties.^7^ Evaluation of the physical properties of orthodontic adhesive could be a useful indicator for the degree of conversion of resin monomer.^10,11^ The degree of conversion of the orthodontic adhesive, fracture resistance and bond strength are expected to increase by increasing the intensity of the light curing device.^12-14^ Although it is required to achieve high bond strength of orthodontic adhesive to resist brackets de-attachment by orthodontic force, the bond strength should be low enough to allow safe removal of the brackets at the end of the treatment with no damage to the enamel.^15^

 Based on the current need of orthodontists for saving the time required for placing the fixed orthodontic appliance and current available literature data, there is a need assess the lowest curing time required for attaching orthodontic brackets to produce a clinically satisfactory bond strength. The bond strength produced by different curing time should also be correlated with the device intensity to provide the orthodontists with a proper recommendation. The degree of conversion can be also indirectly evaluated by measuring the compressive strength of the orthodontic adhesive. Therefore, the current study aimed to measure the shear bond strength of orthodontic adhesives at different curing times and intensities. The compressive strength of orthodontic adhesives was also evaluated.

## Materials and Methods

 Freshly extracted (sound, non-carious, free of previous restorations or defects) human premolars (90 teeth extracted as part of orthodontic treatment) were used in the present study after obtaining ethical approval from the institutional Research Ethic Committee. Teeth were collected after obtaining the patients’ written consent. The teeth were disinfected and kept in distilled water at 4ᴼ C before use. The teeth were mounted with acrylic resin in plastic (PVC) round section tube with buccal surface oriented perpendicular to the cross section of the PVC tube. The buccal surface of the teeth was polished with pumice, washed with water and air-dried. The buccal surface of the teeth was etched with 37% phosphoric acid gel (3M Dental products, Saint Paul, USA) for 15 seconds, washed with water and air-dried for 15 seconds.

Upper first premolar stainless steel orthodontic brackets (Dentaurum, Ispringen, Germany) with surface area of 11.42 mm^2^ were loaded with orthodontic self-adhesive resin (Biofix orthodontic adhesive, Dental Biodynamics, Ibiporã, Brazil), positioned on the middle third of buccal surface of the teeth and light cured (after removing the excess resin around the brackets with dental probe) with VRN-VAFU LED curing light (Guilin Veirun Medical Technology Co., Ltd, Guilin, China). The curing of the adhesive resin was performed at different curing times (1 second, 3 seconds and 5 seconds) using different light curing intensities (1000, 1600 and 2300 mW/cm^2^). The light curing intensity of the LED was measured with radiometer (Guilin Woodpecker Medical Instrument Co., Ltd. Guilin, China) before curing each sample. The curing of the adhesive resin of each bracket was performed from two sides (mesial and distal) for each curing time and intensity. The light curing tip was positioned as close as possible to the adhesive resin with-out touching the brackets. The teeth were kept in distilled water in dark container at 37°C for 24 hours. before measuring the shear bond strength. A custom-made attachment connected to universal testing machine (GESTER International Co., LTD, Quanzhou, China) with a cross head speed of 0.5 mm/min was used to de-attach the orthodontic brackets from the teeth. The dislodgement force and surface area of the brackets were used to calculate the shear bond strength in MPa. After the brackets’ removal, the adhesive remnant was assessed by capturing standardized digital photos for the surface of the teeth examined under a stereomicroscope (OPTIKA, BG, Italy) at × 10 magnification. Image analysis software (ImageJ 1.47b, Java, Wayne Rasband, National Institutes of Health, Bethesda, MD, USA) was used to process the photos and to calculate the ratio of the surface area of the adhesive remnant. Ten samples (N = 10) were tested for each curing time at different intensities.

 For measuring the compressive strength, cylinder-shaped specimens (6 mm height and 4 mm diameter) were produced by incremental (2 mm increment) packing of the orthodontic adhesive resin into a PVC mold. After the placement of the final increment, the specimen was covered with celluloid strip and glass slab to remove the excess material. Each layer was light cured using different curing times and intensities with the shear bond strength experiment. The samples were kept in distilled water in dark container at 37°C for 24 hours. before being fractured using the universal testing machine with a cross head speed of 0.5 mm/min to measure the strength of each sample. Ten samples (n = 10) were tested for each curing time at different intensities.

The results were subjected to statistical analysis using SPSS ver. 11.5.0 (SPSS Inc, Chicago, IL, USA). One-way analysis of variance (ANOVA) was performed followed by multiple comparisons using Tukey’s test. The level of statistical significance was set at *P <*0.05.

## Results

The means and standard deviations of the shear bond strength values are presented in Table 1. The lowest shear bond strength values were recorded with 1000 mW/cm^2^ light curing intensity (at all curing times) in comparison with the other light curing intensities (*P* < 0.05). No significant differences were found between the shear bond strength values of the orthodontic brackets bonded with 1600 mW/cm^2^ light curing intensity with those bonded using 2300 mW/cm^2^ light curing intensity (at 1 and 3 seconds). Increasing the curing time significantly increased the shear bond strength of the orthodontic brackets (*P* < 0.05), except when the curing time was increased from 3 sec to 5 seconds at 1600 mW/cm^2^ light curing intensity.

**Table 1:** Shear bond strength (in MPa)

**Light curing power (mW/cm** ^2^ **)**	**N**	**Light curing time, Mean (SD)**		
		**1 Second**	**3 Seconds**	**5 Seconds**
1000	10	6.4 (1.5) ^A,a^	9.9 (2.4) ^A,b^	12.6 (1.8) ^A,c^
1600	10	9.8 (2.1) ^B,a^	15.2 (1.5) ^B,b^	15.7 (1.6) ^B,b^
2300	10	9.9 (2.6) ^B,a^	15.6 (1.2) ^B,b^	18.2 (2.1) ^C,c^

N, Number of specimens; SD, Standard deviation. Values marked with different uppercase letters (A-C) indicate a significant difference between the different means within the same column. Values marked with different lowercase letters (a-c) indicate a significant difference between the different means within the same row (*P* < 0.05).

The means and standard deviations of the compressive strength values are presented in Table 2. The highest compressive strength values were recorded at 2300 mW/cm^2^ light curing intensity (at all curing times) in comparison with the other light curing intensities (*P* < 0.05). No significant differences were recorded between the compressive strength values of the orthodontic adhesives cured by 1000 mW/cm^2^ and 1600 mW/cm^2^ light curing intensities (except at 3 seconds). Orthodontic adhesives cured with 1 sec (at 1600 and 2300 mW/cm^2^ light curing intensities) had the lowest compressive strength in comparison with orthodontic adhesives cured with 3 and 5 seconds (*P* < 0.05).

**Table 2:** Compressive strength (in MPa)

**Light curing power (mW/cm** ^2^ **)**	**N**	**Light curing time, Mean (SD)**		
		**1 Second**	**3 Seconds**	**5 Seconds**
1000	10	104.2 (11.6) ^A,a^	109.9 (16) ^A,a^	147.4 (26.7) ^A,b^
1600	10	105.9 (15.7) ^A,a^	145.1 (17.5) ^B,b^	152.6 (9.9) ^A,b^
2300	10	130.3 (11.3) ^B,a^	147.1 (12.7) ^B,b^	174 (10.3) ^B,c^

N, Number of specimens; SD, Standard deviation. Values marked with different uppercase letters (A-B) indicate a significant difference between the different means within the same column. Values marked with different lowercase letters (a-c) indicate a significant difference between the different means within the same row (*P* < 0.05).

The means and standard deviations of the ratio of the adhesive remnant values are presented in Table 3. The highest values of the ratio of the adhesive remnants were recorded at 1000 mW/cm^2^ light curing intensities (at all curing times) compared to the other light curing intensities (*P* < 0.05). Increasing the curing times significantly reduced the values of the ratio of the adhesive remnant (*P* < 0.05).

**Table 3:** The ratio of the surface area of the adhesive remnant (surface area of adhesive remnant divided by the total surface area of the adhesion)

**Light curing power (mW/cm** ^2^ **)**	**N**	**Light curing time, Mean (SD)**		
		**1 Second**	**3 Seconds**	**5 Seconds**
1000	10	0.965 (0.19) ^A,a^	0.714 (0.22) ^A,b^	0.709 (0.18) ^A^, ^b^
1600	10	0.756 (0.18) ^B,a^	0.552 (0.22) ^B,b^	0.377 (0.24) ^B,c^
2300	10	0.698 (0.34) ^B,a^	0.536 (0.16) ^B,b^	0.316 (0.27) ^B,c^

N, Number of specimens; SD, Standard deviation. Values marked with different uppercase letters (A-C) indicate a significant difference between the different means within the same column (*P* < 0.05). Values marked with different lowercase letters (a-c) indicate a significant difference between the different means within the same row (*P* < 0.05).

## Discussion

The outcome of this study revealed that increasing the light curing time and the light curing intensity had a positive effect on the shear bond strength of the orthodontic brackets and the compressive strength of the orthodontic adhesive. Increasing the curing light intensity and time exposes the resin to a larger number of photons which improve the polymerization (degree of conversion) by providing higher number of free radicals^16,17^ The mechanical property of resin-based dental material is positively correlated with the light curing time and intensity of the curing light.^18-20^ The degree of the curing of the resin composite depends on the amount of energy (curing time multiplied by the intensity) delivered into the resin composite.^21^ Therefore, curing time can be reduced (to save time and enhance patient’s comfort) but this must be accompanied by an increase in the light curing intensity to keep this energy up to the required level to achieve sufficient polymerization.^22^

The clinically acceptable shear bond strength of orthodontic brackets is 5-8 MPa.^23,24^ The lowest shear bond strength value (6.4 MPa) recorded in the present study is still within the acceptable range. Achieving very high bond strength of orthodontic brackets is not a primary goal in orthodontics as it may put the enamel surface at risk of damage at the end of treatment.^25^

Measuring the mechanical properties of resin composite such as the compressive strength is a useful method of indirectly assessing the degree of conversion and polymerization.^13^ A reduction in the degree of conversion of orthodontic adhesive of about 45% is considered satisfactory.^26^ The lowest compressive strength (104.2 MPa) recorded in the present study, with the shortest curing time and the lowest curing intensity, is about 40% which is less than the highest compressive strength (174 MPa) recorded with the longest curing time and the highest curing intensity. This reduction in the compressive strength (40%) might be accompanied by similar amount of reduction in the degree of conversion and it is still within the acceptable range.

According to the present study, reducing the light curing intensity and/or time increased the ratio of the adhesive remnant (failure at the bracket-adhesive interface). Failure at the bracket-adhesive interface may be attributed to incomplete polymerization caused by short curing time and/or low curing light intensity.^5,27^ On the other hand, increasing the light curing intensity and/or time decreased the ratio of the adhesive remnant (failure at the adhesive-enamel interface). Failure at the adhesive-enamel interface is less desirable in clinical situation as it may be associated with enamel fracture at brackets deboning time.^28,29^ Therefore, the results of adhesive remnants support the reduction in the curing time suggested by the present study.

The present study has some limitations because the bond strength was measured after short period of time (24 hours) and the factors involved within the oral environment have not been considered.^30,31^ Furthermore, the brackets debonding was performed under a speed of 0.5 mm/minute which is slower than speed of brackets debonding under actual clinical conditions.^31^

## Conclusion

Increasing the curing time and\or the curing intensity has a positive influence on the bond strength and compressive strength of the orthodontic adhesive. On the other hand, and within the limitations of the current study, it might be possible to suggest reducing the curing time of orthodontic adhesive to 1 second at curing intensity of 2300 mW/cm^2^.

## Acknowledgments

 This study was supported by the college of Dentistry at the University of Mosul / Iraq.

## Competing Interests

 No competing interests.

## Ethical Approval

 This research was approved by the Research Ethics Committee of the college of Dentistry at the University of Mosul. Approval Reference Number: UoM.Dent/H.DM.80/21

## Funding

 The study is self-funded.
